# Differential Expression of LHR and FSHR in Canine Mammary Tumors: Correlation with Malignancy and Spay Status

**DOI:** 10.3390/vetsci12050496

**Published:** 2025-05-19

**Authors:** Yujue Li, Siying Wang, Jiaxuan Gao, Xuerou Tu, Shihui Yu, Yang Liu, Zhaoxia Zhang, Yuan Cui, Yougang Zhong

**Affiliations:** 1 Department of Clinical Veterinary Medicine, College of Veterinary Medicine, China Agricultural University, Beijing 100193, China; 2 Clinical Laboratory Diagnostic Center, China Agricultural University Veterinary Teaching Hospital, College of Veterinary Medicine, China Agricultural University, Beijing 100193, China

**Keywords:** canine mammary tumors, luteinizing hormone receptor, follicle-stimulating hormone receptor, spay status, tumor malignancy

## Abstract

Canine mammary tumors (CMTs) constitute one of the most prevalent malignancies in female canines, whereas the functional involvement of gonadotropin receptors—luteinizing hormone receptor (LHR) and follicle-stimulating hormone receptor (FSHR)—in these neoplasms remains uncharacterized. This study investigated LHR and FSHR expression in 79 CMTs and 14 normal mammary tissues collected from 59 female dogs (ethical approval AW82903202-2-3), using immunohistochemistry (IHC) and quantitative real-time PCR, specifically examining their association with tumor malignancy and spay status. Relative to normal mammary tissue, CMTs exhibited statistically significant downregulation of both LHR and FSHR transcripts and corresponding proteins (*p* < 0.01), with expression progressively decreasing as tumor malignancy increased, and with grade 3 tumors showing almost undetectable levels. Notably, spayed dogs displayed higher LHR levels in tumors (*p* < 0.05), whereas intact dogs showed higher FSHR levels than spayed dogs. These findings suggest that the expression of LHR and FSHR in CMTs may correlate with tumor malignancy and be influenced by the spay status. Our findings provide the first evidence of gonadotropin receptor dysregulation in CMTs, offering novel insights into their molecular mechanisms and potential applications in hormone-related diagnostic or therapeutic strategies.

## Simple Summary

Canine mammary tumors represent a significant oncological concern in female dogs. Our investigation revealed a marked downregulation of two key reproductive regulators—luteinizing hormone receptor and follicle-stimulating hormone receptor—in canine mammary tumors relative to normal mammary tissues. The most aggressive tumors showed the lowest levels of these receptors. Interestingly, spayed dogs had higher luteinizing hormone receptor levels in their tumors, while intact dogs showed more follicle-stimulating hormone receptor. These findings underscore the crucial roles of both receptors in tumorigenesis and disease progression. By linking receptor levels to tumor behavior and spay status, our research may enable veterinarians to identify high-risk cases earlier and guide novel therapeutic strategies, such as hormone-based therapies, ultimately improving survival rates in dogs with mammary tumors.

## 1. Introduction

Canine mammary tumors (CMTs) represent the predominant neoplastic disease in female dogs, constituting 50–70% of all tumors in intact females [1,2,3]. Approximately 50% of CMTs are malignant, posing significant clinical challenges due to their metastatic potential. Early studies have demonstrated that ovariohysterectomy (OHE), the cornerstone strategy for CMT prevention, markedly reduces their incidence by eliminating ovarian hormone exposure [4]. However, recent evidence indicates that OHE fails to significantly decrease the recurrence risk in diagnosed CMT cases [5], and spayed dogs exhibit higher rates of malignant mammary epithelial tumors compared with their intact counterparts [6]. This paradox suggests that OHE may modulate tumor progression through non-ovarian hormone-dependent pathways, although the molecular mechanisms remain uncharacterized.

Ovarian excision-induced gonadotropin elevation may participate in regulating malignant phenotypes of CMTs. OHE causes loss of ovarian hormone feedback in the hypothalamic–pituitary–gonadal (HPG) axis, resulting in sustained supraphysiological levels of luteinizing hormone (LH) and follicle-stimulating hormone (FSH) [7]. Although LH and FSH primarily regulate reproductive functions, their receptors are widely expressed in non-gonadal tissues, including the skin [8], bladder/urethra [9,10], musculoskeletal tissues [11], lymphocytes/thymus [12], mast cells [13], and prostate [14]. Notably, LH receptor (LHR) and FSHR are aberrantly expressed in non-reproductive tumors of spayed dogs, such as lymphoma [12], splenic hemangiosarcoma [15,16], mastocytoma [13], prostate cancer [17], and transitional cell carcinoma [18], and correlate with increased tumor incidence post-gonadectomy. Current research on the hormonal regulation of CMTs predominantly focuses on estrogen/progesterone pathways [19,20,21]. While post-OHE hypergonadotropinism has been linked to certain non-reproductive tumors [12,13,15,16,17,18], systematic investigations into LHR/FSHR expression patterns in CMTs and their correlations with tumor malignancy or spay status remain absent, significantly impeding risk assessment and therapeutic development based on gonadotropin signaling.

Based on this, we hypothesize that differential expression of LHR and FSHR exists in CMT tissues, with expression levels correlating with tumor malignancy and spay status. To test this hypothesis, we performed the first systematic analysis of LHR/FSHR transcript and protein expression profiles in CMT specimens, employing histological grading comparisons and spay status subgroup analyses. This study aims to establish the baseline expression characteristics of LHR/FSHR in CMTs, elucidate their correlations with clinicopathological parameters, and provide essential foundational data for subsequent mechanistic investigations. Our findings advance the molecular characterization of CMTs and inform optimization of sterilization strategies and targeted therapeutic development.

## 2. Materials and Methods

### 2.1. Canine Mammary Samples

This study included 59 female dogs undergoing mammary tumor surgery at the China Agricultural University Veterinary Teaching Hospital between 2023 and 2024. These samples included 79 mammary tumor tissues (as some dogs had multiple tumors) and 14 normal mammary tissues. Normal mammary specimens were procured from female dogs that underwent unilateral mastectomy, with the sampling regions strictly ≥2 mammary glands away from the tumor edge.

The case selection criteria were as follows: All dogs underwent clinical inspection and palpation to assess tumor quantity, location, and size, followed by complete surgical excision. Postoperative histopathological examination by pathologists at our institution confirmed primary mammary tumors according to Goldschmidt’s modified classification system for canine mammary tumors [22]. Complete demographic data (breed, age, spay status) were recorded. Distant metastasis was screened via thoracic radiography or CT, while lymphatic invasion was confirmed by fine-needle aspiration cytology and/or postoperative histopathology. The exclusion criteria included concurrent systemic malignancies, preoperative chemotherapy, or incomplete clinical records. All dogs were clinically staged using the TNM system. Tumors were classified according to the revised criteria proposed by Goldschmidt et al. (2011) [22], and malignant epithelial tumors were graded using the Nottingham histological grading system [23]. Samples were categorized into the following groups: (1) control group (normal mammary tissues), (2) benign tumors, and (3) malignant tumors (stratified into histological grades 1, 2, and 3).

### 2.2. Sample Processing

The samples included 14 normal mammary tissues and 79 mammary tumor tissues. All samples were fixed with 4% paraformaldehyde solution for immunohistochemistry (IHC). Due to the limited availability of fresh tumor samples, only a subset was preserved in liquid nitrogen for RNA extraction, including 14 normal mammary tissues and 37 mammary tumor tissues. The distribution of the samples processed for immunohistochemistry and RNA extraction is detailed in Supplementary Table S1.

### 2.3. Immunohistochemical Staining

Following 10% neutral buffered formalin fixation, mammary tissue specimens underwent paraffin embedding using standard histological protocols. Paraffin-embedded tissue sections (4 μm) were dewaxed in xylene, rehydrated through a graded alcohol series (100%, 95%, 80%, 70%), subjected to heat-mediated epitope recovery in sodium citrate buffer via 20 min of boiling at 100 °C, and subsequently cooled to ambient temperature. Endogenous peroxidase activity was blocked using a peroxidase inhibitor (ZSGB-BIO, Beijing, China). To reduce nonspecific binding, tissue sections were incubated with 10% caprine serum at ambient temperature (30 min). Immunostaining was performed using polyclonal rabbit antibodies against LHR (1:100 dilution, Boster, Wuhan, China) and FSHR (1:150 dilution, Bioss, Beijing, China) through 16 h of cold exposure at 4 °C. Subsequent detection employed goat anti-rabbit IgG (H+L) conjugated to horseradish peroxidase (ZSGB-BIO, Beijing, China) under optimized thermal conditions (37 °C, 30 min). The DAB reaction (1:20 dilution, ZSGB-BIO, Beijing, China) was monitored microscopically under ambient conditions (2–3 min), with termination timed upon visual confirmation of optimal staining intensity, followed by hematoxylin counterstaining for 6 min. Following a tap water rinse, the sections underwent an ascending ethanol series, xylene clearance, and resin-based mounting for microscopic analysis. Canine normal ovarian tissues, demonstrating physiological LHR and FSHR expression, served as intrinsic positive controls. Assay specificity was validated through parallel negative controls, where primary antibodies were substituted with PBS, and representative images of these controls are provided in Supplementary Figure S1, with the controls demonstrating the absence of nonspecific staining.

The immunostaining results for LHR and FSHR in mammary samples were semi-quantitatively evaluated [24,25]. Under high magnification, at least five randomly selected fields were examined, and a minimum of 500 epithelial cells were quantified to ascertain the percentage of positive cells (PS). PS was scored using the following stratified criteria: 0 (less than 14%), 1 (15–24%), 2 (25–64%), and 3 (more than 65%). The intensity of immunolabeling (IS) was quantitatively assessed using Image Pro Plus 6.0.0 and stratified into four levels: 0 (negative), 1 (weak), 2 (moderate), and 3 (strong). The final immunohistochemical histoscore (HS) was calculated as the product of PS and IS.

### 2.4. RNA Extraction and qRT-PCR Analysis

RNA extraction was performed on 37 mammary tumors and 14 normal mammary specimens using a TRIzol-based RNA isolation protocol. Quantitative analysis of RNA concentration and purity was conducted through spectrophotometry measurements (NanoDrop 2000, Thermo Scientific, Waltham, MA, USA). Subsequent reverse-transcription reactions were processed with equivalent template quantities (500 ng of RNA input) through enzymatic conversion using a commercial cDNA synthesis system (GenStar BioSolutions, Zibo, China). Quantitative real-time PCR (qRT-PCR) was conducted in a 20 μL reaction set following the manufacturer’s guidelines (Yeasen Biotech, Shanghai, China). The qRT-PCR amplification protocol involved an initial DNA denaturation phase of 2 min at 95 °C, with subsequent thermal cycling consisting of 40 repetitions, each including a denaturation step at 95 °C for 10 s and an annealing phase at 60 °C maintained for 34 s. Table 1 presents the specific primer sequences. The relative quantification of mRNA expression levels was determined using the comparative threshold cycle method (2^−ΔΔCt^). Glyceraldehyde 3-phosphate dehydrogenase (*GAPDH*) functioned as the endogenous reference gene for data normalization. To ensure methodological reproducibility, all experiments were performed in triplicate with independently prepared biological replicates, incorporating both technical and experimental controls.

### 2.5. Statistical Analysis

Statistical analyses were performed using JASP 0.19 (University of Amsterdam, Amsterdam, The Netherlands) and Microsoft Excel 2016 (Microsoft Corporation, Redmond, WA, USA). Data presentation was standardized according to variable types: categorical variables were expressed as percentages (%), while continuous variables were reported as the mean ± SEM. The distributional characteristics were rigorously evaluated through Shapiro–Wilk normality testing and Levene’s variance homogeneity assessment. For two-group comparisons, parametric analysis using Student’s *t*-test was conducted when both assumptions were satisfied; otherwise, the nonparametric Mann–Whitney U test was employed. In multigroup analyses, parametric datasets underwent one-way ANOVA with either Tukey’s or Dunnett’s post hoc testing, whereas nonparametric distributions were analyzed using the Kruskal–Wallis H test followed by Dunn’s multiple comparison correction. The significance levels are indicated as * *p* < 0.05, ** *p* < 0.01, and *** *p* < 0.001.

## 3. Results

### 3.1. Animal Data and Histopathological Characteristics of Tumors

This study involved 59 female dogs diagnosed with mammary tumors, of which 72.9% (43/59) were intact and 27.1% (16/59) were spayed. The sample encompassed 13 different breeds, with Poodles being the most common, accounting for 55.9% (33/59) of the dogs. The mean age of the dogs was 10.29 ± 0.37 years (mean ± SEM), with intact dogs averaging 9.79 ± 0.40 years and spayed dogs 11.66 ± 0.77 years. The spayed dogs were significantly older than the intact dogs (*p* < 0.05). The majority of the tumors were classified as T1 stage (<3 cm), accounting for 50.8% (30/59) of cases. A significantly higher proportion of T3 stage tumors (>5 cm) was observed in spayed dogs (31.3%, 5/16) compared to intact dogs (15.0%, 6/43). Lymph node invasion occurred in 10.2% (6/59) of cases, with spayed dogs showing a higher incidence (12.5%, 1/16) than intact dogs (7.0%, 3/43). Distant metastases were identified in 8.5% (5/59) of cases at diagnosis, demonstrating a consistent trend of elevated frequency in spayed dogs (12.5%, 2/16) relative to intact dogs (7.0%, 3/43). Based on the TNM clinical staging system, the cohort distribution was as follows: Stage I (47.5%, 28/59), Stage II (23.7%, 14/59), Stage III (11.9%, 7/59), Stage IV (8.5%, 5/59), and Stage V (8.5%, 5/59). Advanced-stage tumors (Stages III–V) were significantly more prevalent in spayed dogs (37.5%, 6/16) than in intact dogs (25.6%, 11/43), indicating accelerated tumor progression in spayed canines. Detailed data are presented in Table 2. Histopathological analysis classified the tumors into benign and malignant categories. The proportions of benign tumors, grade 1 malignant tumors, grade 2 malignant tumors, and grade 3 malignant tumors were 13.6% (8/59), 44.1% (26/59), 30.5% (18/59), and 11.9% (7/59), respectively. For dogs with multiple tumors, the highest malignancy grade was used for classification. The proportion of malignant tumors was slightly higher in spayed dogs (87.5%) compared to intact dogs (86.0%).

A total of 79 mammary tumors from 59 dogs were analyzed, comprising 21 benign tumors and 58 malignant tumors. Among the benign tumors, three subtypes were identified, with benign mixed tumors being the most common (*n* = 10). The malignant tumors encompassed 12 subtypes, with mixed carcinoma being the most frequent (*n* = 15). Detailed classifications are presented in Table 3.

### 3.2. Immunohistochemical Characterization of LHR and FSHR

**Figure 1:**
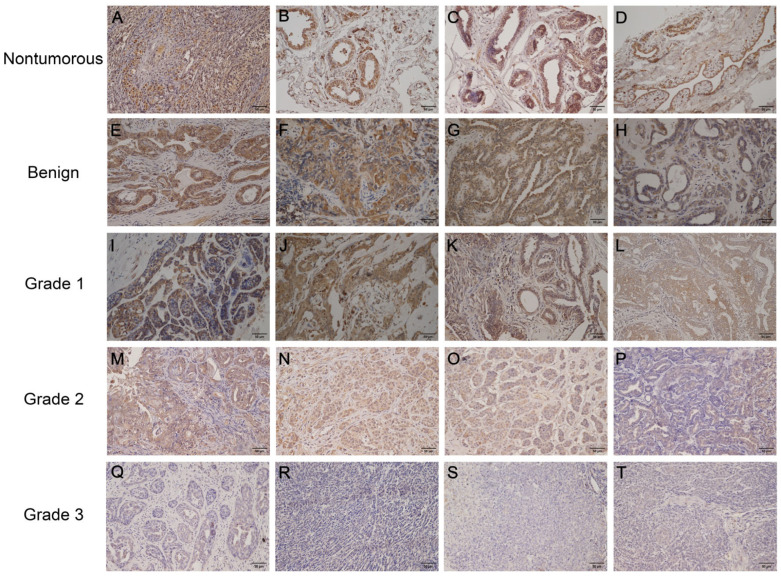
Immunohistochemical analysis of luteinizing hormone receptor (LHR) expression in canine mammary tissues. Positive control (canine ovarian tissue) (**A**); normal mammary tissues (**B**–**D**): cytoplasmic immunostaining intensity was strong in epithelial cells (histoscore [HS]: 9); benign tumors (**E**–**H**): cytoplasmic immunostaining intensity ranged from moderate to weak in neoplastic epithelial cells (HS: 6, 4, 3, 2); grade 1 malignant tumors (**I**–**L**): cytoplasmic immunostaining intensity decreased from moderate to weak (HS: 4, 3, 2, 1); grade 2 malignant tumors (**M**–**P**): weak cytoplasmic immunostaining (HS: 2, 2, 1, 1); grade 3 malignant tumors (**Q**–**T**): no detectable immunostaining signals were identified in the cytoplasm of neoplastic epithelial cells (HS: 0 in all samples). Scale bar: 50 μm.

**Figure 2:**
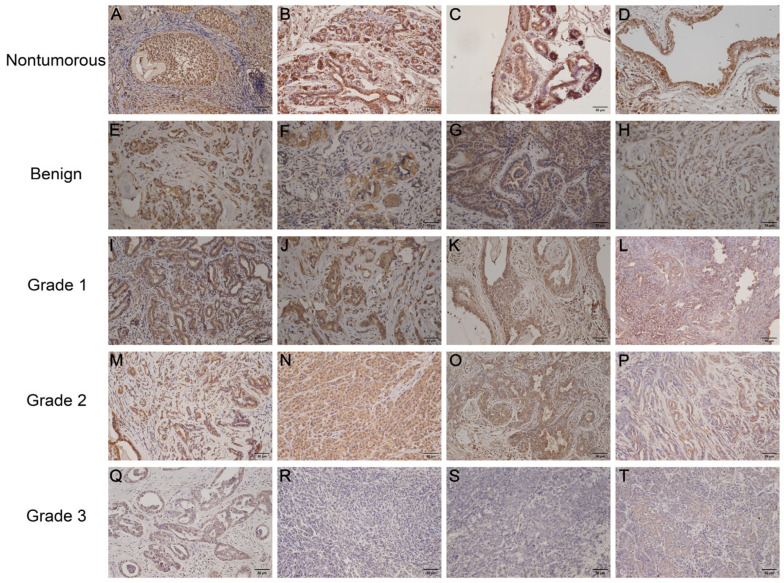
Immunohistochemical analysis of follicle-stimulating hormone receptor (FSHR) expression in canine mammary tissues. Positive control (canine ovarian tissue) (**A**); normal mammary tissues (**B**–**D**): cytoplasmic immunostaining intensity was strong in epithelial cells (HS: 9); benign tumors (**E**–**H**): cytoplasmic immunostaining intensity ranged from moderate to weak in neoplastic epithelial cells (HS: 6, 4, 3, 2); grade 1 malignant tumors (**I**–**L**): cytoplasmic immunostaining intensity decreased from moderate to weak (HS: 4, 3, 2, 2); grade 2 malignant tumors (**M**–**P**): cytoplasmic immunostaining intensity progressively decreased (HS: 4, 3, 2, 1); grade 3 malignant tumors (**Q**–**T**): cytoplasmic immunostaining intensity ranged from weak to none (HS: 1, 0, 0, 0). Scale bar: 50 μm.

**Figure 3:**
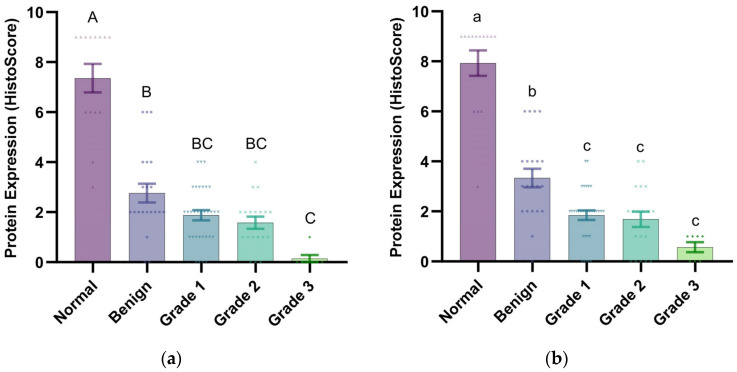
Expression levels of LHR and FSHR proteins in canine mammary tissues. (**a**) LHR histoscore: notable distinctions were identified among normal tissues, benign tumors, and malignant tumors (grades 1–3) (*p* < 0.01, uppercase letters A/B/C); (**b**) FSHR histoscore: notable distinctions were identified among normal tissues, benign tumors, and malignant tumors (grades 1–3) (*p* < 0.05, lowercase letters a/b/c). Data points represent individual samples, with distinct marker shapes indicating tissue types: normal mammary tissues (▲, *n* = 14), benign mammary tumors (●, *n* = 21), grade 1 mammary tumors (▼, *n* = 32), grade 2 mammary tumors (✖, *n* = 19), and grade 3 mammary tumors (◆, *n* = 7).

Immunohistochemical staining employing a semi-quantitative histoscoring system (Table 4; HS = PS × IS) was conducted to evaluate gonadotropin receptor (LHR/FSHR) distribution patterns across canine mammary tissue specimens, comprising 14 histologically normal controls and 79 neoplastic samples. Canine ovarian tissue served as a positive control, exhibiting strong expression of both LHR and FSHR (Figure 1A and Figure 2A). No nonspecific staining was observed in the negative control specimens (Supplementary Figure S1). In normal mammary tissues, LHR and FSHR exhibited marked expression in the cytosolic regions of the mammary epithelium (Figure 1B–D and Figure 2B–D). Conversely, mammary tumor tissues displayed heterogeneous expression of LHR and FSHR in the cytosolic regions of the neoplastic epithelium, with levels ranging from negative to strong expression (Figure 1E–T and Figure 2E–T). Furthermore, compared to tumor tissues, normal mammary tissues had dramatically higher expression levels of LHR and FSHR (*p* < 0.001 and *p* < 0.001, respectively). The PS, IS, and HS values were consistently higher in normal mammary tissues (Table 5). Notably, as the malignancy grade increased and differentiation decreased, the protein expression of LHR and FSHR significantly decreased, with minimal to no expression observed in grade 3 mammary tumors (Table 5). Significant differences in LHR and FSHR expression were detected between grade 3 malignant tumors and benign mammary tumors (Figure 3; *p* < 0.01 and *p* < 0.05, respectively). Additionally, FSHR expression showed significant differences between benign and malignant tumors at various grades (Figure 3b; *p* < 0.05).

Considering the effects of ovariectomy on canine LH and FSH levels, we performed an additional analysis of LHR and FSHR expression in dogs with varying spay statuses. The protein expression of LHR in mammary tumor tissues was elevated in spayed dogs compared to intact female dogs (Table 6), particularly in the HS (*p* < 0.05). Interestingly, the protein expression trend of FSHR was opposite to that of LHR, as FSHR expression increased in the mammary tumors of intact female dogs, although no significant difference was observed (Table 6).

### 3.3. qRT-PCR Validation of LHR and FSHR Expression Patterns

**Figure 4:**
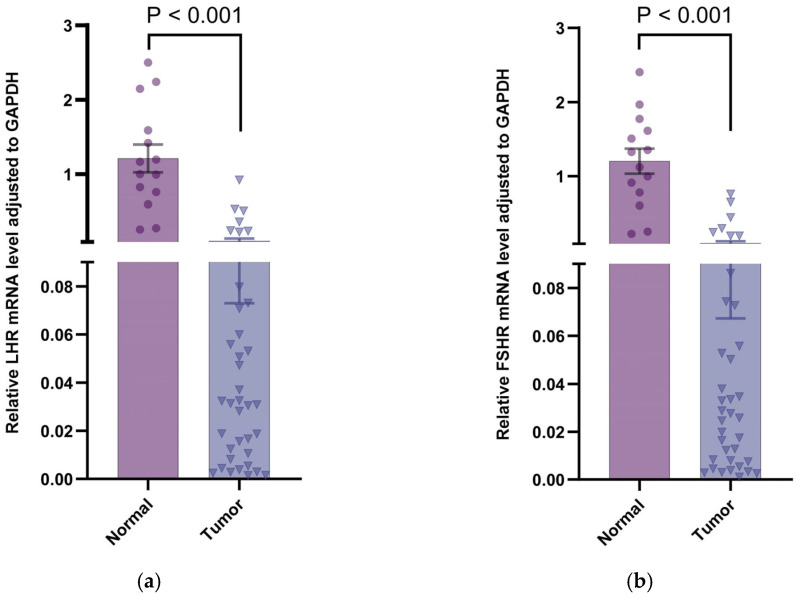
Relative mRNA expression of gonadotropin receptor in canine mammary tissues. (**a**) *LHR* mRNA expression normalized to *GAPDH*: a marked reduction in expression was observed in tumor tissues relative to normal counterparts (*p* < 0.001); (**b**) *FSHR* mRNA expression normalized to *GAPDH*: a marked reduction in expression was observed in tumor tissues relative to normal counterparts (*p* < 0.001). Data points represent individual samples, with distinct marker shapes indicating tissue types: normal mammary tissues (●, *n* = 14), mammary tumor tissues (▼, *n* = 37).

**Figure 5:**
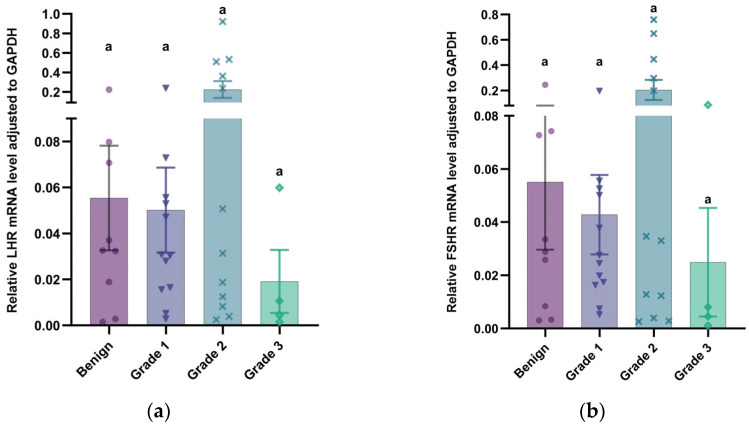
Relative mRNA expression of gonadotropin receptor in canine mammary tumors. (**a**) *LHR* mRNA expression normalized to *GAPDH* in benign tumors and malignant mammary tumors (grades 1–3). Statistical comparisons between any groups showed nonsignificant divergence (*p* > 0.05); (**b**) *FSHR* mRNA expression normalized to *GAPDH* in benign tumors and malignant mammary tumors (grades 1–3). Statistical comparisons between the groups showed nonsignificant divergence (*p* > 0.05, lowercase letter a). Data points represent individual samples, with distinct marker shapes indicating tissue types: benign mammary tumors (●, *n* = 9), grade 1 mammary tumors (▼, *n* = 12), grade 2 mammary tumors (✖, *n* = 12), and grade 3 mammary tumors (◆, *n* = 4).

Transcriptional dynamics validation via qRT-PCR confirmed detectable expression of *LHR* and *FSHR* mRNA transcripts in all mammary specimens analyzed, including 14 physiological mammary controls and 37 mammary tumor tissues from canines (Table 7). Compared to normal mammary tissues, the transcript levels of *LHR* and *FSHR* were markedly diminished in mammary tumor tissues (Figure 4; *p* < 0.001 and *p* < 0.001, respectively). Additionally, the results demonstrated that the transcript quantification of *LHR* and *FSHR* was lowest in grade 3 mammary tumor tissues, consistent with the immunohistochemistry results. However, statistical comparisons between the groups showed nonsignificant divergence (Figure 5; *p* > 0.05).

## 4. Discussion

At present, gonadectomy constitutes the mainstay strategy for managing dog populations and has been shown to significantly lower the prevalence of reproduction-related pathologies [4,28,29,30]. However, accumulating evidence suggests that the abnormal elevation of LH and FSH levels is associated with the progression of various neoplastic diseases [31,32,33,34,35]. Despite this, the existing literature lacks investigation into the changes in LHR and FSHR expression in CMTs. This study analyzes the clinical information and histological diagnostic results of 59 cases of CMTs, assessing their association with LHR/FSHR expression patterns at both the protein and transcriptional levels. The results indicate potential involvement of LHR and FSHR in the tumorigenesis and progression of CMTs.

Estrogen is essential for controlling the reproductive capabilities of female animals and exerts a significant influence on the metabolic processes, growth, and development of the organism. Previous studies have demonstrated that estrogen promotes mammary tumor cell proliferation via apoptosis inhibition [36]. In this study, 72.9% (51/69) of canine mammary tumor cases occurred in intact female dogs, which may correlate with prolonged endogenous hormone exposure. However, clinical analysis of 59 canine mammary tumor cases revealed that spayed dogs exhibited larger tumor volumes, higher rates of lymph node invasion and distant metastasis, and a significantly increased proportion of advanced-stage (Stages III–V) cases. This may indicate enhanced clinical invasiveness of mammary tumors in spayed dogs. Additionally, spayed dogs demonstrated an elevated risk of malignant tumors compared to intact dogs, consistent with previous reports indicating that spaying reduces the overall incidence of mammary tumors but increases the proportion of malignant subtypes [6]. This paradoxical phenomenon implies that spaying may exert dual mechanisms on canine mammary tumorigenesis: it reduces the overall tumor incidence by eliminating gonadal-derived estrogen, while simultaneously activating non-sex-hormone-dependent oncogenic pathways through endocrine remodeling.

Notably, the dramatic hormonal fluctuations induced by spaying constitute a core mechanism underlying this complexity. Post-ovariectomy estrogen withdrawal disrupts negative feedback regulation, leading to a sharp elevation of serum LH and FSH levels, which can surge to dozens of times the pre-spaying concentrations [37,38,39,40]. Growing evidence indicates that LHR and FSHR are widely distributed in non-reproductive organs and certain tumor tissues [34,35], where they are associated with tumor diseases such as lymphoma and mastocytoma [12,13]. LHR and FSHR expression has been confirmed in human mammary tumor tissues and cell lines [24,25,41]. Similarly, we detected LHR and FSHR expression in canine mammary tumor tissues, with distinct expression patterns correlated with spay status: LHR expression was upregulated in tumors from spayed dogs, whereas FSHR expression showed a downward trend. This differential receptor expression may critically explain the enhanced aggressiveness of mammary tumors in spayed dogs. Human breast cancer studies have demonstrated that the LH–LHR signaling axis promotes tumor cell adhesion, migration, and stromal invasion by activating cortactin/N-WASP phosphorylation cascades [42], and the observed LHR upregulation in canine models may suggest analogous pro-metastatic mechanisms. These findings collectively point to an under-recognized pathophysiological process wherein spaying-induced hypergonadotropinemia may drive malignant progression through LHR/FSHR-mediated, non-sex-hormone-dependent pathways that reshape the tumor microenvironment.

Previous studies have shown that LH/FSH signaling through LHR/FSHR activates G protein-coupled ROCK–moesin and FAK–paxillin axes, significantly enhancing invasive pseudopod formation and transendothelial migration in breast cancer cells [32,42]. In CMTs, we observed dynamic expression patterns of LHR and FSHR: compared to healthy mammary tissues, both gene transcription and protein expression of LHR and FSHR were markedly downregulated in CMTs, with further reductions accompanying increased tumor malignancy and decreased differentiation. Notably, grade 3 mammary tumors exhibited near-complete loss of receptor expression. This expression pattern is highly consistent with human breast cancer studies, where gonadotropin receptors maintain baseline expression in normal mammary epithelial cells but undergo receptor silencing in poorly differentiated or invasive tumors [24,25,41]. This malignancy-dependent receptor attenuation may reflect an endogenous defense mechanism during advanced tumor progression, whereby downregulation of gonadotropin receptors weakens hormone-driven pro-metastatic signaling. However, such compensatory regulation appears insufficient to counteract established invasive phenotypes. These findings suggest that LHR/FSHR expression levels may serve as critical molecular markers for evaluating CMT biological behavior, with their dynamic changes providing key insights into gonadotropin signaling modes across tumor developmental stages.

While this study elucidates associations between LHR/FSHR and malignancy or spay status in CMTs, several limitations warrant consideration. At the sample level, although 59 dogs and 79 tumor tissues were included, the small sample size of grade 3 tumors (*n* = 4) and imbalanced distribution across malignancy grades may affect the statistical power and correlation accuracy. Normal mammary tissue samples were obtained from dogs undergoing unilateral mastectomy, and although the sampling areas were histopathologically validated and maintained a safe distance from the tumor margins, potential disease-related influences on receptor expression cannot be entirely excluded. Methodologically, immunohistochemical semi-quantitative scoring carries inherent subjectivity despite multi-field counting and software assistance. Regarding the population and external validity, all samples originated from a single hospital, predominantly comprising local breeds (e.g., Poodles, Bichons), with limited genetic diversity and absence of long-term follow-up data to assess the impact of receptor expression on recurrence or prognosis. Mechanistically, this study focused on LHR/FSHR expression without evaluating serum hormone levels, downstream signaling pathways, interactions with other hormone receptors, or molecular mechanisms underlying spaying-induced receptor changes, limiting biological interpretation to observational levels. Future studies should expand the sample sizes, implement multicenter collaborations, adopt objective detection technologies, incorporate additional variables, and conduct functional experiments to validate and extend these findings, thereby advancing the understanding of CMT pathogenesis and the clinical implications of spaying.

## 5. Conclusions

In conclusion, our findings confirm the expression of LHR and FSHR in CMTs. Additionally, the upregulation of LHR expression following OHE may be a key factor influencing the severity of CMTs. However, the specific mechanisms underlying this effect remain to be elucidated.

## Figures and Tables

**Table 1:** Primer sequences utilized for the measurement of mRNA synthesis via real-time RT-PCR.

Gene Symbol	Gene Name	Primer Sequence (5′→3′)	Amplicon Length (bp)	Accession Number	Reference
*GAPDH*	Glyceraldehyde 3-phosphate dehydrogenase	F: CTGAACGGGAAGCTCACTGG	220	NP 001003142.2	Adapted from [26]
		R: CGTCGAAGGTGGAAGAGTGG			
*LHR*	Luteinizing hormone receptor	F: AAACCAAAGGCCAGTATTATAACCA	78	XP 038534822.1	Adapted from [8]
		R: AGTGAAAAAGCCAGCTGCACTAC			
*FSHR*	Follicle-stimulating hormone receptor	F: ATTAGCATCCTGGCCATCAC	122	XM_014117520.1	Adapted from [27]
		R: CCAATGCAGAGATCAGCAAA			

**Table 2:** Information of dogs with mammary tumors.

	Total (*n* = 59)	Intact (*n* = 43)	Spayed (*n* = 16)
Age of case (y)			
Range	4.00–16.00	5.00–16.00	4.00–15.00
Mean ± SEM	10.29 ± 0.37	9.79 ± 0.40	11.66 ± 0.77
Breed			
Poodle	33 (55.9%)	27 (62.8%)	6 (37.5%)
Mixed breed	9 (15.3%)	6 (14.0%)	3 (18.8%)
Bichon Frise	5 (8.5%)	5 (11.6%)	0
Pomeranian	2 (3.4%)	1 (2.3%)	1 (6.3%)
Siberian Husky	2 (3.4%)	0	2 (12.5%)
Border Collie	1 (1.7%)	0	1 (6.3%)
German Shepherd	1 (1.7%)	1 (2.3%)	0
Golden Retriever	1 (1.7%)	1 (2.3%)	0
Corgi	1 (1.7%)	1 (2.3%)	0
Labrador Retriever	1 (1.7%)	1 (2.3%)	0
Rottweiler	1 (1.7%)	0	1 (6.3%)
Samoyed	1 (1.7%)	0	1 (6.3%)
Japanese Spitz	1 (1.7%)	0	1 (6.3%)
Tumor size ^1^			
T1	30 (50.8%)	21 (48.8%)	9 (56.3%)
T2	18 (30.5%)	16 (37.2%)	2 (12.5%)
T3	11 (18.6%)	6 (15.0%)	5 (31.3%)
Lymphatic invasion ^2^			
N0	53 (89.8%)	39 (90.7%)	14 (87.5%)
N1	6 (10.2%)	4 (9.3%)	2 (12.5%)
Distant metastases ^3^			
M0	54 (91.5%)	40 (93.0%)	14 (87.5%)
M1	5 (8.5%)	3 (7.0%)	2 (12.5%)
Clinical stage ^4^			
I	28 (47.5%)	19 (44.2%)	9 (56.3%)
II	14 (23.7%)	13 (30.2%)	1 (6.3%)
III	7 (11.9%)	4 (9.3%)	3 (18.8%)
IV	5 (8.5%)	4 (9.3%)	1 (6.3%)
V	5 (8.5%)	3 (7.0%)	2 (12.5%)
Histological type			
Benign	8 (13.6%)	6 (14.0%)	2 (12.5%)
Malignant	51 (86.4%)	37 (86.0%)	14 (87.5%)
Grade 1	26 (44.1%)	18 (41.9%)	8 (50.0%)
Grade 2	18 (30.5%)	14 (32.6%)	4 (25.0%)
Grade 3	7 (11.9%)	5 (11.6%)	2 (12.5%)

^1^ Tumor dimensional classification (T1: maximum diameter < 3 cm; T2: 3–5 cm; T3: exceeding 5 cm). ^2^ Regional lymphatic spread status (N0: no metastatic involvement; N1: lymph node involvement). ^3^ Distant metastases (M0: no detectable distant lesions; M1: radiologically verified metastases). ^4^ Clinical staging according to TNM system: Stage I: localized tumor (T1), no nodal/distant spread; Stage II: moderate tumor extent (T2), N0M0; Stage III: advanced local invasion (T3), N0M0; Stage IV: regional nodal metastasis (N1), any T, M0; Stage V: distant metastasis (M1), any T/N.

**Table 3:** Histological subtypes of tumor samples.

Histologic Subtype	Total (*n* = 79)	Intact (*n* = 53)	Spayed (*n* = 26)
Benign tumor	21 (26.6%)	12 (22.6%)	9 (34.6%)
Benign mixed tumor	10 (12.7%)	6 (11.3%)	4 (15.4%)
Complex adenoma	8 (10.1%)	6 (11.3%)	2 (7.7%)
Simple adenoma	3 (3.8%)	0	3 (11.5%)
Malignant tumor	58 (73.4%)	41 (77.4%)	17 (65.4%)
Mixed carcinoma	15 (19.0%)	13 (24.5%)	2 (7.7%)
Complex carcinoma	12 (15.2%)	10 (18.9%)	2 (7.7%)
Simple carcinoma	7 (8.9%)	5 (9.4%)	2 (7.8%)
Intraductal papillary carcinoma	7 (8.9%)	5 (9.4%)	2 (7.9%)
Tubular carcinoma	5 (6.3%)	3 (5.7%)	2 (7.10%)
Ductal carcinoma	4 (5.1%)	2 (3.8%)	2 (7.11%)
Carcinoma with malignant myoepithelioma	2 (2.5%)	1 (1.9%)	1 (3.8%)
Solid carcinoma	2 (2.5%)	1 (1.9%)	1 (3.8%)
Malignant spindle cell tumor	1 (1.3%)	0	1 (3.8%)
Carcinoma arising in a benign mixed tumor	1 (1.3%)	0	1 (3.8%)
Comedocarcinoma	1 (1.3%)	0	1 (3.8%)
Micropapillary invasive carcinoma	1 (1.3%)	1 (1.9%)	0

**Table 4:** Histopathological classification, spay status of bitch, and malignancy grade of immunohistochemistry samples.

Histological Type	Number of Immunohistochemistry Samples		
	Total [Grade 1, 2, 3]	Intact [Grade 1, 2, 3]	Spayed [Grade 1, 2, 3]
Benign tumor	21	12	9
Benign mixed tumor	10	6	4
Complex adenoma	8	6	2
Simple adenoma	3	0	3
Malignant tumor	58	41	17
Mixed carcinoma	15 (11, 4, 0)	13 (9, 4)	2 (2, 0, 0)
Complex carcinoma	12 (7, 5, 0)	10 (5, 5, 0)	2( 2, 0, 0)
Simple carcinoma	7 (2, 3, 2)	5 (0, 3, 2)	2 (2, 0, 0)
Intraductal papillary carcinoma	7 (6, 1, 0)	5 (4, 1, 0)	2 (2, 0, 0)
Tubular carcinoma	5 (2, 3, 0)	3 (1, 2, 0)	2 (1, 1, 0)
Ductal carcinoma	4 (3, 1, 0)	2 (2, 0, 0)	2 (1, 1, 0)
Carcinoma with malignant myoepithelioma	2 (0, 0, 2)	1 (0, 0, 1)	1 (0, 0, 1)
Solid carcinoma	2 (0, 1, 1)	1 (0, 0, 1)	1 (0, 1, 0)
Malignant spindle cell tumor	1 (0, 1, 0)	0	1 (0, 1, 0)
Carcinoma arising in a benign mixed tumor	1 (1, 0, 0)	0	1 (1, 0, 0)
Comedocarcinoma	1 (0, 0, 1)	0	1 (0, 0, 1)
Micropapillary invasive carcinoma	1 (0, 0, 1)	1 (0, 0, 1)	0

**Table 5:** LHR/FSHR immunohistochemical expression in neoplastic and normal mammary tissues.

	Number of Samples	PS ^1^ (LHR)	IS ^2^ (LHR)	HS ^3^ (LHR)	PS (FSHR)	IS (FSHR)	HS (FSHR)
Normal mammary tissue	14	64.06 ± 6.09	2.79 ± 0.11	7.36 ± 0.57	65.24 ± 6.74	3.00 ± 0.00	7.93 ± 0.51
Mammary tumor	79	37.12 ± 3.01	1.23 ± 0.06	1.89 ± 0.16	38.20 ± 2.88	1.48 ± 0.07	2.09 ± 0.17
Benign tumor	21	44.91 ± 6.32	1.62 ± 0.11	2.76 ± 0.38	47.10 ± 5.18	1.67 ± 0.13	3.33 ± 0.37
Malignant tumor	58	34.30 ± 3.37	1.16 ± 0.06	1.57 ± 0.16	34.97 ± 3.37	1.41 ± 0.09	1.64 ± 0.16
Grade 1	32	41.77 ± 4.77	1.25 ± 0.08	1.88 ± 0.20	40.80 ± 4.43	1.31 ± 0.09	1.84 ± 0.19
Grade 2	19	30.45 ± 4.87	1.16 ± 0.09	1.58 ± 0.25	33.53 ± 6.12	1.68 ± 0.19	1.68 ± 0.31
Grade 3	7	10.61 ± 4.37	0.71 ± 0.18	0.14 ± 0.14	12.23 ± 3.62	1.14 ± 0.14	0.57 ± 0.20

^1^ PS, the percentage of positive cells; ^2^ IS, the intensity of immunolabeling; ^3^ HS, immunohistochemical histoscore; HS = PS × IS.

**Table 6:** LHR/FSHR immunohistochemistry in mammary tumors across spay status (PS, IS, HS).

	Intact (a)	Spayed (b)	*p* (a vs. b)
Number of samples	53	26	
PS (LHR)	35.41 ± 3.82	40.62 ± 4.84	0.298
IS (LHR)	1.25 ± 0.07	1.35 ± 0.10	0.478
HS (LHR)	1.66 ± 0.19	2.35 ± 0.28	0.033
PS (FSHR)	40.80 ± 3.65	32.90 ± 4.52	0.286
IS (FSHR)	1.51 ± 0.10	1.423 ± 0.10	0.905
HS (FSHR)	2.13 ± 0.21	2.00 ± 0.31	0.456

**Table 7:** Histopathological classification, spay status of bitch, and malignancy grade of qRT-PCR samples.

Histological Type	Number of qRT-PCR Samples		
	Total [Grade 1, 2, 3]	Intact [Grade 1, 2, 3]	Spayed [Grade 1, 2, 3]
Benign tumor	9	9	0
Benign mixed tumor	5	5	0
Complex adenoma	4	4	0
Malignant tumor	28 (12, 12, 4)	24 (11, 10, 3)	4 (1, 2, 1)
Mixed carcinoma	9 (6, 3, 0)	9 (6, 3, 0)	0
Complex carcinoma	5 (3, 2, 0)	5 (3, 2, 0)	0
Simple carcinoma	5 (1, 2, 2)	4 (0, 2, 2)	1 (1, 0, 0)
Intraductal papillary carcinoma	3 (2, 1, 0)	3 (2, 1, 0)	0
Tubular carcinoma	3 (0, 3, 0)	2 (0, 2, 0)	1 (0, 1, 0)
Carcinoma with malignant myoepithelioma	1 (0, 0, 1)	1 (0, 0, 1)	0
Solid carcinoma	1 (0, 1, 0)	0	1 (0, 1, 0)
Comedocarcinoma	1 (0, 0, 1)	0	1 (0, 0, 1)

## Data Availability

Data are contained within the article and Supplementary Materials.

## Supplementary Materials

The following supporting information can be downloaded at: https://www.mdpi.com/article/10.3390/vetsci12050496/s1, Supplementary Table S1: Clinicopathological characteristics and sample testing profile of canine mammary tumor cases (complete immunohistochemistry with partial qRT-PCR coverage). Supplementary Figure S1: Negative control for LHR/FSHR immunohistochemistry (PBS substitution for primary antibody).

## Abbreviations

The following abbreviations are used in this manuscript:

CMTs	Canine mammary tumors
LHR	Luteinizing hormone receptor
FSHR	Follicle-stimulating hormone receptor
OHE	Ovariohysterectomy
HPG	Hypothalamic–pituitary–gonadal
qRT-PCR	Quantitative real-time PCR
GAPDH	Glyceraldehyde 3-phosphate dehydrogenase
PS	The percentage of positive cells
IS	The intensity of immunolabeling
HS	Immunohistochemical histoscore
