# Supplementary material for: Differential Expression of LHR and FSHR in Canine Mammary Tumors: Correlation with Malignancy and Spay Status
Source: Vet Sci. 2025 May 19;12(5):496. doi: 10.3390/vetsci12050496 (PMC12116059; doi:10.3390/vetsci12050496)
Supplement: Supplementary file 1 [file vetsci-12-00496-s001.zip › vetsci-3579247-supplementary.pdf]

Supplementary Table S1: Clinicopathological characteristics and sample testing profile of canine mammary tumor cases (complete immunohistochemistry with partial qRT-PCR coverage)

Case	Spay Status	Age (y)	Breed	Tumor size[1]	Lymphatic invasion[2]	Distant metastases[3]	Clinical stage[4]	Tumor	Histological type	Immunohistochemistry samples	qRT-PCR samples
1	Intact	13	Poodle	T3	N0	M1	Ⅴ	(1)	Simple carcinoma, Grade 2	√	√
								(2)	Benign mixed tumor	√	√
								(3)	Intraductal papillary carcinoma, Grade 1	√	√
								(4)	Complex carcinoma, Grade 2	√	√
2	Intact	8	German shepherd	T3	N0	M0	Ⅲ	(1)	Intraductal papillary carcinoma, Grade 1	√	
								(2)	Mixed carcinoma, Grade 1	√	
3	Intact	10	Poodle	T2	N1	M0	Ⅳ	(1)	Simple carcinoma, Grade 3	√	√
4	Intact	11	Poodle	T2	N0	M0	Ⅱ	(1)	Intraductal papillary carcinoma, Grade 1	√	√
5	Intact	8	Poodle	T1	N0	M0	Ⅰ	(1)	Complex carcinoma, Grade 1	√	√
6	Intact	5	Corgi	T1	N0	M0	Ⅰ	(1)	Simple carcinoma, Grade 2	√	√
7	Intact	12	Poodle	T2	N0	M0	Ⅱ	(1)	Mixed carcinoma, Grade 2	√	√
8	Intact	10	Mixed breed	T3	N0	M0	Ⅲ	(1)	Carcinoma with Malignant myoepithelioma, Grade 3	√	√
9	Intact	9	Mixed breed	T2	N0	M0	Ⅱ	(1)	Complex carcinoma, Grade 1	√	√
10	Intact	13	Bichon Frise	T2	N0	M0	Ⅱ	(1)	Mixed carcinoma, Grade 1	√	√
								(2)	Mixed carcinoma, Grade 1	√	
11	Intact	8	Golden Retriever	T2	N0	M0	Ⅱ	(1)	Mixed carcinoma, Grade 1	√	√
12	Intact	13	Mixed breed	T2	N0	M1	Ⅴ	(1)	Simple carcinoma, Grade 3	√	√
13	Intact	7.5	Poodle	T1	N0	M0	Ⅰ	(1)	Mixed carcinoma, Grade 1	√	√
14	Intact	16	Mixed breed	T1	N0	M0	Ⅰ	(1)	Complex adenoma	√	√
15	Intact	10	Poodle	T1	N0	M0	Ⅰ	(1)	Benign mixed tumor	√	√
								(2)	Complex adenoma	√	√

16	Intact	9	Mixed breed	T1	N0	M0	Ⅰ	(1)	Complex adenoma	√	√
17	Intact	14	Poodle	T1	N0	M0	Ⅰ	(1)	Complex carcinoma, Grade 2	√	√
								(2)	Complex adenoma	√	√
18	Spayed	10	Poodle	T1	N0	M0	Ⅰ	(1)	Complex adenoma	√	
19	Intact	8.8	Poodle	T2	N0	M0	Ⅱ	(1)	Complex adenoma	√	
20	Spayed	11	Poodle	T1	N0	M0	Ⅰ	(1)	Simple carcinoma, Grade 1	√	
								(2)	Intraductal papillary carcinoma, Grade 1	√	
								(3)	Mixed carcinoma, Grade 1	√	
								(4)	Complex adenoma	√	
21	Intact	11.5	Bichon Frise	T1	N0	M0	Ⅰ	(1)	Ductal carcinoma, Grade 1	√	
								(2)	Complex adenoma	√	
22	Spayed	8	Poodle	T1	N0	M0	Ⅰ	(1)	Simple adenoma	√	
23	Spayed	14	Mixed breed	T1	N0	M0	Ⅰ	(1)	Complex carcinoma, Grade 1	√	
								(2)	Simple adenoma	√	
24	Spayed	13	Poodle	T2	N1	M0	Ⅳ	(1)	Malignant spindle cell tumor, Grade 2	√	
								(2)	Benign mixed tumor	√	
								(3)	Simple adenoma	√	
25	Intact	16	Poodle	T1	N0	M0	Ⅰ	(1)	Benign mixed tumor	√	
26	Intact	10	Poodle	T2	N0	M0	Ⅱ	(1)	Benign mixed tumor	√	√
27	Intact	6	Bichon Frise	T2	N0	M0	Ⅱ	(1)	Benign mixed tumor	√	√
28	Intact	7	Poodle	T1	N0	M0	Ⅰ	(1)	Mixed carcinoma, Grade 1	√	√
								(2)	Benign mixed tumor	√	√
29	Spayed	13.5	Poodle	T1	N0	M0	Ⅰ	(1)	Complex carcinoma, Grade 1	√	
								(2)	Benign mixed tumor	√	
30	Spayed	15	Japanese Spitz	T2	N0	M0	Ⅱ	(1)	Mixed carcinoma, Grade 1	√	
								(2)	Benign mixed tumor	√	
31	Spayed	15	Pomeranian	T1	N0	M0	Ⅰ	(1)	Carcinoma arising in a benign mixed tumor, Grade 1	√	
								(2)	Tubular carcinoma, Grade 1	√	
								(3)	Benign mixed tumor	√	
32	Intact	6	Poodle	T1	N0	M0	Ⅰ	(1)	Tubular carcinoma	√	
33	Intact	9	Labrador retriever	T2	N0	M0	Ⅱ	(1)	Ductal carcinoma, Grade 1	√	

34	Spayed	13	Siberian Husky	T1	N0	M0	I	(1)	Ductal carcinoma, Grade 1	√	
35	Intact	8	Poodle	T1	N0	M0	I	(1)	Intraductal papillary carcinoma, Grade 1	√	
36	Spayed	7	Rottweiler	T3	N0	M0	III	(1)	Intraductal papillary carcinoma, Grade 1	√	
37	Spayed	4	Poodle	T1	N0	M0	I	(1)	Simple carcinoma, Grade 1	√	√
38	Intact	11	Poodle	T3	N0	M0	III	(1)	Mixed carcinoma, Grade 2	√	
								(2)	Complex carcinoma, Grade 1	√	
39	Intact	13	Poodle	T2	N0	M0	II	(1)	Complex carcinoma, Grade 1	√	
40	Intact	8	Bichon Frise	T1	N0	M0	I	(1)	Complex carcinoma, Grade 1	√	√
41	Intact	8	Pomeranian	T1	N0	M0	I	(1)	Mixed carcinoma, Grade 1	√	
42	Intact	7	Poodle	T2	N0	M0	II	(1)	Mixed carcinoma, Grade 1	√	√
43	Intact	12	Poodle	T3	N0	M1	V	(1)	Mixed carcinoma, Grade 1	√	√
44	Intact	12	Poodle	T1	N1	M0	IV	(1)	Tubular carcinoma, Grade 2	√	√
45	Spayed	13	Mixed breed	T1	N0	M0	I	(1)	Tubular carcinoma, Grade 2	√	√
46	Intact	9	Bichon Frise	T2	N0	M0	II	(1)	Tubular carcinoma, Grade 2	√	√
47	Spayed	14	Border Collie	T3	N0	M0	III	(1)	Ductal carcinoma, Grade 2	√	
48	Intact	8	Poodle	T2	N0	M0	II	(1)	Intraductal papillary carcinoma, Grade 2	√	√
49	Intact	8	Poodle	T1	N0	M0	I	(1)	Simple carcinoma, Grade 2	√	
50	Intact	12	Mixed breed	T1	N0	M0	I	(1)	Complex carcinoma, Grade 2	√	
51	Intact	5	Poodle	T1	N0	M0	I	(1)	Complex carcinoma, Grade 2	√	
52	Intact	11	Poodle	T3	N0	M0	III	(1)	Complex carcinoma, Grade 2	√	
53	Intact	8	Poodle	T1	N0	M0	I	(1)	Mixed carcinoma, Grade 2	√	√
54	Intact	9	Poodle	T2	N1	M0	IV	(1)	Mixed carcinoma, Grade 2	√	√
55	Spayed	11	Siberian Husky	T3	N0	M1	V	(1)	Solid carcinoma, Grade 2	√	√

56	Intact	10	Poodle	T1	N0	M0	Ⅰ	(1)	Solid carcinoma, Grade 3	√	
57	Intact	11	Poodle	T1	N1	M0	Ⅳ	(1)	Micropapillary invasive carcinoma, Grade 3	√	
58	Spayed	13	Mixed breed	T3	N1	M1	Ⅴ	(1)	Carcinoma with Malignant myoepithelioma, Grade 3	√	
59	Spayed	12	Samoyed	T3	N0	M0	Ⅲ	(1)	Comedocarcinoma, Grade 3	√	√

[1] Tumor dimensional classification (T1: maximum diameter < 3 cm; T2: 3–5 cm; T3: exceeding 5 cm). [2] Regional lymphatic spread status (N0: no metastatic involvement; N1: lymph-node involvement). [3] Distant metastases (M0: no detectable distant lesions; M1: radiologically verified metastases). [4] Clinical staging according to TNM system, Stage I: Localized tumor (T1), no nodal/distant spread; Stage II: Moderate tumor extent (T2), N0M0; Stage III: Advanced local invasion (T3), N0M0; Stage IV: Regional nodal metastasis (N1), any T, M0; Stage V: Distant metastasis (M1), any T/N. √: Performed.

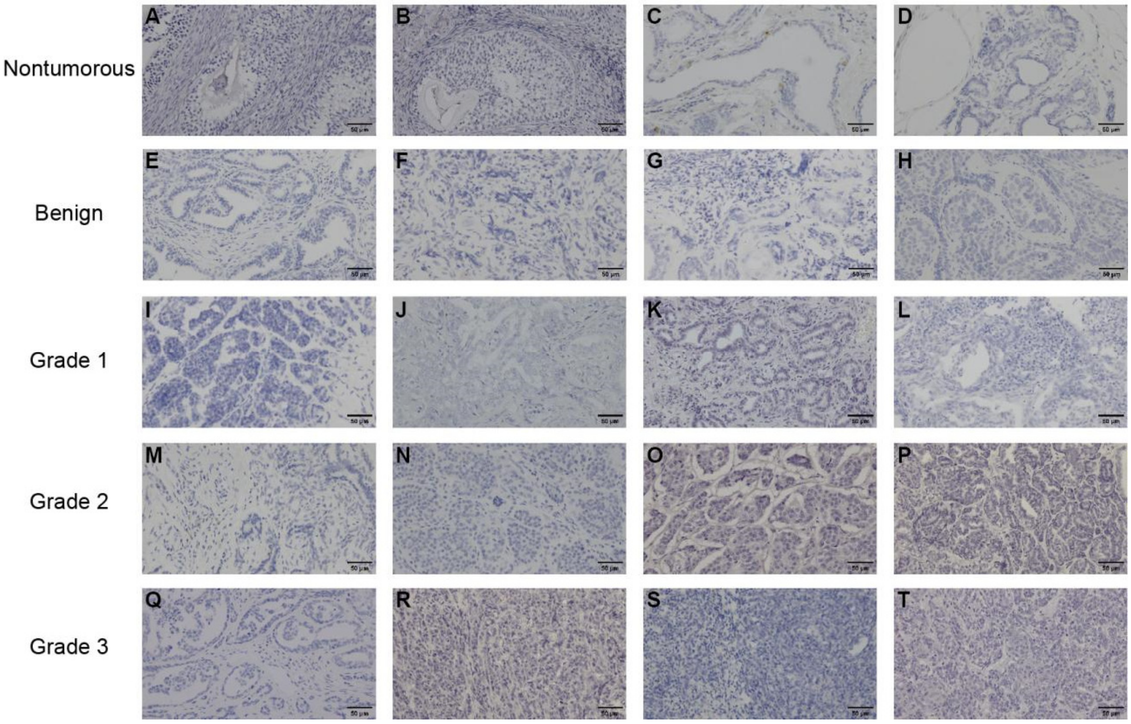

Supplementary Figure S1: Negative control for LHR/FSHR immunohistochemistry (PBS substitution for primary antibody). Canine ovarian tissue (A, B); Normal canine mammary tissue (C, D); Benign tumors (E–H); Grade 1 malignant tumors (I–L); Grade 2 malignant tumors (M–P); Grade 3 malignant tumors (Q–T). No specific staining signals were detected in any tissue regions (DAB chromogen with hematoxylin counterstaining), indicating no nonspecific primary antibody binding. Scale bar: 50 μm.
